# Virulence Regulation in *Borrelia burgdorferi*

**DOI:** 10.3390/microorganisms13092183

**Published:** 2025-09-18

**Authors:** Sierra George, Zhiming Ouyang

**Affiliations:** Department of Molecular Medicine, University of South Florida, 12901 Bruce B Downs Blvd, MDC 07, Tampa, FL 33612, USA; sierrageorge@usf.edu

**Keywords:** Lyme disease, *Borrelia burgdorferi*, gene regulation, alternative sigma factor, DNA binding protein, molecular microbiology

## Abstract

*Borrelia burgdorferi,* the causative agent of Lyme disease, is the most common vector-borne disease in the United States. Compared with other bacterial pathogens, *B. burgdorferi* has many unique features. For instance, its highly segmented genome was predicted to encode very few proteins directly dedicated to gene expression regulation. Yet, the spirochete continuously reprograms its transcriptome and proteome to promote survival and pathogenesis as spirochetes traverse the enzootic lifecycle between ticks and mammals. Signal sensing systems, a unique alternative sigma factor cascade, and multi-functional regulators work in concert to coordinate virulence gene expression under different tick and mammal environments. In this review, we have summarized recent advances in gene regulation in *B. burgdorferi*.

## 1. Introduction

Lyme disease is the most common vector-borne disease in the United States. The causative agent of Lyme disease was initially discovered in 1982 when the spirochete bacterium *Borrelia burgdorferi* (also known as *Borreliella burgdorferi*) *sensu stricto* was isolated from *Ixodes* ticks [1]. In the years since its discovery, numerous groups have worked to define, characterize, and understand how this unique pathogen survives, adapts, and causes disease.

*B. burgdorferi* lacks genes required for many cellular biosynthetic reactions and does not encode common virulence factors (e.g., toxins) used by other pathogenic bacteria [2]. Instead, the spirochete relies on tightly coordinated gene expression to adapt to and survive in ticks and small mammals. Despite a limited repertoire of regulatory proteins, gene regulation in *B. burgdorferi* is complex, often consisting of multiple layers of regulation at the transcriptional, post-transcriptional, and post-translational levels. Precisely regulating the expression of unique virulence factors allows the spirochete to be transmitted between the tick vector and mammalian hosts and cause incidental human disease. Therefore, untangling gene regulatory networks in *B. burgdorferi* has become a critical field of study.

In this review, we summarize the current knowledge of *B. burgdorferi* genome organization, how the pathogen senses and responds to environmental changes, and mechanisms of gene regulation, primarily the RpoN-RpoS alternative sigma factor pathway and other transcriptional regulators.

## 2. *Borrelia burgdorferi*: The Causative Agent of Lyme Disease

### 2.1. The Enzootic Lifecycle

*B. burgdorferi* survives in an enzootic lifecycle where it is transmitted between a tick vector and mammalian hosts [3,4,5,6]. The cycle begins when *Ixodes scapularis* larvae acquire the bacterium from an infected small mammal, commonly the white-footed mouse (*Peromyscus* spp.), during a bloodmeal (i.e., acquisition phase). Fed larvae molt into unfed nymphs (i.e., molting phase) before taking a second bloodmeal and subsequently transmitting the spirochetes to a naïve host (i.e., transmission phase), again the white-footed mouse or another small mammal [7,8,9]. Fed nymphs molt into adults. Female adult ticks take a third and final bloodmeal on a larger mammal such as the white-tailed deer before mating and laying eggs. It is widely accepted that there is no transovarial transmission of *B. burgdorferi* [10]. In addition to the white-footed mouse and the white-tailed deer, other avian and reptilian species also play important roles during the enzootic lifecycle [7,8,9,11]. Humans are not an essential part of the *B. burgdorferi* lifecycle but are incidentally infected due to the generalist feeding behavior of *I. scapularis* ticks [12]. Ticks may feed up to seven days on a mammalian host, and spirochete transmission occurs after 72 h of tick attachment [13,14].

The survival of *B. burgdorferi* within an enzootic lifecycle has been largely attributed to its ability to tightly regulate gene expression. Specifically, changes in gene expression allow the spirochete to express proteins essential for survival at various stages such as acquisition, tick molting, migration of the pathogen from the midgut to the salivary glands, tick–mammal transmission, evasion of host immune responses, and binding host components for dissemination in mammals. Investigating these mechanisms in vivo is often difficult due to low bacterial numbers in ticks and host tissues; however, a DMC (dialysis membrane chamber) model was developed to obtain host-adapted spirochetes for studying in vivo gene expression regulation [15]. Moreover, in vitro conditions have been widely used to mimic, at least partially, critical phases of the enzootic lifecycle. For example, shifts in temperature, pH, and carbon source of growth media can induce signature transcriptomic changes observed in vivo [3,16,17].

### 2.2. Genome Organization

*B. burgdorferi* was the first spirochete pathogen to have its complete genome sequenced [2]. Compared with other bacterial species, it has the most highly segmented genome, consisting of a linear chromosome nearly one megabase in size and approximately 20 additional linear and circular plasmids; plasmid content varies slightly depending on the strain of *B. burgdorferi*. Across the genome, G + C content is low and ranges from 23% to 32% on the chromosome and all plasmids [2]. In total, the *B. burgdorferi* genome encodes only 1283 genes, far fewer than other bacterial pathogens such as *Escherichia coli* which encodes ~4300 genes [2]. Interestingly, the vast majority of *B. burgdorferi*-annotated genes encode proteins of unknown function. The chromosome is believed to be well conserved amongst *B. burgdorferi* strains and encodes genes involved in basic biological processes such as DNA replication, transcription, translation, and metabolism [2]. Several *B. burgdorferi* plasmids are essential for spirochete survival during various phases of the enzootic lifecycle including tick acquisition, tick molting, transmission to the mammalian host, or during mammalian infection. Specifically, *cp*26, *lp17*, and *lp54* are found in all Lyme disease strains [18,19,20,21]. *cp*26 is required for growth in vitro and encodes genes for chitobiose import, the telomere resolvase ResT, and the outer surface protein C (OspC) essential for early mammalian infection [22,23]. *lp17* encodes BBD18, a repressor of the alternative sigma factor RpoS, and a small RNA (SR0726), that are important for tick acquisition and mammalian infection, respectively [24,25,26,27,28,29,30]. *lp54* encodes several genes essential for tick colonization (such as outer surface proteins OspA and OspB) and mammalian infection (such as decorin-binding proteins DbpA and DbpB) [31,32,33,34]. Plasmids *lp25*, *lp36*, *lp28-1*, and *lp28-4* are also important for tick–mammal transmission and/or spirochete survival in the mammalian host [35,36,37,38,39,40,41].

## 3. Environmental Sensing by Two-Component Systems

*B. burgdorferi* regulates its gene expression in response to various tick and mammal factors including temperature, pH, cell density, growth phase, external and internal metabolites, and redox status, to name a few [42,43,44,45,46,47]. For example, temperate shifts from 23 °C to 37 °C and pH adjustments from 7.6 to 6.8 partially mimic the midgut environment in feeding ticks during tick–mammal transmission. Mid-logarithmic growth and stationary-phase growth partially mimics the changes in bacterial replication and cell density during tick feeding, when spirochetes exhibit rapid replication prior to transmission. During tick molting, spirochetes experience a period of starvation compared to the nutrient rich environments of the fed tick and mammalian host. Combined, the status of the spirochete’s environment triggers a physiological response, which shapes the transcriptomic and proteomic landscapes for survival under the given condition. However, many questions remain open regarding how the bacterium senses the environment to induce gene expression changes. To date, two-component systems are the best studied signal transduction systems in *B. burgdorferi* gene regulation.

In many bacterial pathogens, two-component systems (TCSs), consisting of a histidine kinase (HK) and response regulator (RR), act as a signaling cascade, allowing bacteria to sense, respond, and adapt to environmental cues [48,49,50]. Generally, the HK contains an N-terminal input domain that extends outside of the bacterial cell membrane and senses a specific signal such as a metabolite, osmotic pressure, or pH. Upon stimulation of the input domain, the HK C-terminal domain auto-phosphorylates a conserved histidine residue. The phosphate is subsequently transferred to an aspartate residue on the corresponding cytoplasmic RR. Phosphorylation of the RR alters the protein conformation and, in turn, its ability to bind DNA and regulate gene transcription [51,52]. Given that the HK sensing domain is usually specific to a single external signal, it is not uncommon for bacteria to encode multiple TCSs to respond and adapt to a variety of environmental changes [53,54,55]. Interestingly, the *B. burgdorferi* genome encodes only two TCS: HK1-Rrp1 and HK2-Rrp2 [2].

### 3.1. HK1-Rrp1

HK1-Rrp1 is essential for spirochete survival during tick feeding, but not in mammalian hosts [56,57,58]. Structurally, HK1 consists of a periplasmic sensor domain and histidine kinase core. Rrp1 differs from traditional-TCS RRs in that it contains the phosphorylation receiver domain, but lacks a DNA-binding domain and instead possesses a GGDEF domain for diguanylate cyclase activity [59]. Upon sensing a yet-unidentified ligand, it is presumed that HK1 auto-phosphorylates and transfers the phosphate to the Rrp1 receiver domain, as observed in other TCSs. The activation of Rrp1 diguanylate cyclase activity results in the production of bis-(3’-5’)-cyclic dimeric GMP (c-di-GMP), a small secondary messenger that is capable of binding target proteins to alter their DNA binding activity [58,59,60]. In *B. burgdorferi*, c-di-GMP has been shown to bind a DNA/RNA binding protein, PlzA, to regulate genes involved in carbohydrate utilization, motility, virulence, and host adaptation [61,62,63,64,65,66,67,68]. Currently, PlzA is the only known PilZ domain protein in all Lyme disease spirochetes capable of binding c-di-GMP.

### 3.2. HK2-Rrp2

The second *B. burgdorferi* TCS, HK2-Rrp2, is also unique from traditional TCSs. First, the entire histidine kinase (HK2) is located in the cytoplasm, suggesting it responds to internal rather than external signal(s); to date, the signal activating HK2-Rrp2 remains elusive [56,69]. Second, the RR (Rrp2), but not HK2, is essential for mammalian infectivity [70,71]. HK2 does, however, contribute to infectivity via natural tick-bite suggesting an important role for the protein during the enzootic lifecycle [72]. Finally, HK2 is not required for the phosphorylation of its cognate RR (Rrp2), indicating that another yet-to-be-identified phosphate donor serves to activate Rrp2 [72]. In fact, the overexpression of HK2 results in decreased levels of phosphorylated Rrp2, suggesting that HK2 functions instead as a phosphatase to de-phosphorylate Rrp2 [73].

Upon phosphorylation, Rrp2 acts as the bacterial enhancer binding protein (bEBP) for the alternative sigma factor, RpoN, thus positively controlling the expression of the alternative sigma factor RpoS [69,72,74,75,76,77]. Additional lines of evidence, however, suggest that Rrp2 plays an independent role outside of the RpoN-RpoS pathway. First, RpoN, but not Rrp2, is easily inactivated and does not contribute to bacterial growth [78]. Second, constitutive expression of OspC, an outer surface protein regulated by RpoS, in an *rrp2* point mutant, does not rescue the ability of the point mutant to infect the mammalian host [70]. Moreover, Rrp2 is essential for spirochete growth, independent of its function as a bEBP [79,80,81]. Additionally, global sequencing analysis identified more than 100 genes differentially expressed in an *rrp2* point mutant, independent of the RpoN-RpoS pathway, including virulence-associated genes and heat-shock protein-encoding genes [70,74]. However, studies investigating the targets of Rrp2 have been performed under in vitro conditions and have been limited by an inability to generate a full Rrp2 mutant. Rather, mutants have been generated by introducing point mutations to inactivate the functional domains of the protein. Subsequent studies are warranted to determine the essential nature of Rrp2, independent of the RpoN-RpoS pathway.

Although HK1-Rrp1 and HK2-Rrp2 two-component systems are required during different phases of the enzootic lifecycle, there is increasing evidence for crosstalk between these two pathways. For example, MCP5, a methyl-accepting chemotaxis protein is regulated by both HK1-Rrp1 and HK2-Rrp2 [82]. *mlp* family genes activated by the RpoN-RpoS pathway are also differentially expressed in *rrp1* mutant strains [60]. Furthermore, the expression of *rpoS* and RpoS-regulated genes such as *ospC* are lower in both *rrp1* and *rrp2* mutant strains, suggesting that RpoS and the RpoS regulon may be regulated by both two-component systems; an early finding consistent with more recent evidence that c-di-GMP, the product of Rrp1 activation, regulates the RpoS regulon [60,65,67,83]. However, the precise relationship between HK1-Rrp1 and HK2-Rrp2 and how they overlap to regulate *B. burgdorferi* transmission and host adaptation remain to be elucidated.

## 4. Regulators of Gene Expression

### 4.1. RpoS, the Central Alternative Sigma Factor

To adapt to changing environments, bacteria must quickly alter their transcriptome and express or repress subsets of genes that promote survival under a given condition. Transcription initiation is tightly regulated and requires the coordination of the RNA polymerase (RNAP) enzyme at a gene promoter, i.e., a DNA sequence upstream of the transcriptional start site (TSS) [84,85,86]. RNAP holoenzyme consists of four core polypeptides: two identical α chains, a β chain, a β’ chain, and a ω chain. RNAP promoter specificity is determined by a fifth dissociable subunit, the σ (sigma) factor [87,88]. Each class of sigma factor allows RNAP to recognize select promoter sequences and express specific groups of genes in response to environmental signals. For example, housekeeping genes involved in basic biological processes including transcription, translation, metabolism, and exponential growth are generally recognized by RpoD (σ^70^) at a −35/−10 promoter. This consensus sequence motif, TTGACA and TATAAT, is centered at 35 and 10 base pairs upstream of the gene TSS, respectively. In addition to σ^70^, bacteria typically encode alternative sigma factors such as σ^32^ (RpoH), σ^54^ (RpoN), σ^S^ (RpoS), or σ^28^ (RpoF), which direct RNAP to recognize and transcribe genes required for response to heat shock, nitrogen assimilation, general stress, or chemotaxis, respectively [89,90,91]. However, *B. burgdorferi* encodes only RpoD, RpoN, and RpoS sigma factors [2].

As aforementioned, RpoS functions in most bacteria to regulate general stress response genes involved in adaptation to temperature, pH, or starvation. In *E. coli*, for example, RpoS expression increases at the stationary phase of growth and responds to multiple stimuli to provide broad stress protection [92,93]. Similarly, in *Pseudomonas aeruginosa*, RpoS responds to heat, oxidative stress, and osmotic shock [94,95]. In *B. burgdorferi*, however, RpoS does not coordinate general stress responses and instead regulates genes required for spirochete survival and virulence in the mammalian host or tick vector [76,77]. RpoS not only activates *B. burgdorferi* genes required for tick–mammal transmission and mammalian infectivity but it also represses many σ^70^-dependent genes important for colonization and persistence in ticks. This unique function has led to RpoS’s designation as a “gatekeeper” of *B. burgdorferi* gene expression [96]. Upon tick feeding during the acquisition phase, RpoS is turned “off” and remains “off” as *B. burgdorferi* resides in the tick midgut during molting. The absence of RpoS during acquisition and intermolt allows for the transcription of genes essential for *B. burgdorferi* survival in the unfed tick, including *ospA*, encoding a protein required for the attachment of spirochetes to the tick midgut [32]. After molting, when unfed ticks take a second bloodmeal during the transmission phase, RpoS is turned “on” to active the transcription of genes required for transmission and survival in the mammalian host including *ospC*, which contributes to the evasion of host complement-mediated killing [97,98,99]. RpoS remains “on” during mammalian infection to activate genes such as *dbpA* and *dbpB*, encoding adhesins that promote spirochete dissemination in the mammalian host [34,97,100].

#### 4.1.1. Activation of *rpoS* Expression

The activation of *rpoS* transcription in *B. burgdorferi* is complex, requiring the alternative sigma factor, RpoN, and several additional activators [16]. Specifically, RpoN recognizes a conserved −24/−12 promoter sequence upstream of the *rpoS* TSS. Of note, *rpoS* is the only currently known gene in the *B. burgdorferi* genome with the −24/−12 promoter sequence for RpoN recognition [101]. It is also the only gene whose expression was experimentally confirmed to be regulated by RpoN in *B. burgdorferi* [16,74,75,97]. As with typical σ^54^ sigma factors, RpoN activation requires the bEBP Rrp2 in *B. burgdorferi* [72,75]. The Rrp2 activation domain is activated by phosphorylation and then associates with RpoN to promote open complex formation [71,102]. In addition to Rrp2, a second *trans*-acting DNA binding protein, BosR (*Borrelia* oxidative stress regulator), is required for *rpoS* transcription [103,104,105]. BosR is a member of the ferric uptake regulator (Fur) family of direct transcriptional regulators [46,106]. In other bacteria, the binding of Fur protein to DNA results in the repression of genes involved in iron homeostasis; however, in *B. burgdorferi,* BosR does not regulate iron homeostasis, possesses an atypical DNA binding motif, and functions as a dual-functional regulatory protein for gene expression activation and repression [105,107,108,109,110,111,112]. Specifically, BosR binding to the *rpoS* promoter via a DNA sequence termed the ‘BosR Box’ activates *rpoS* transcription [105]. More recently, BosR was also proposed to bind the *rpoS* 5’ mRNA end and promote stability, leading to increased RpoS protein levels [113]. Therefore, in contrast to canonical RpoN sigma factors, which require a single bEBP, the transcription of *rpoS* by RpoN requires two *trans*-acting activators, Rrp2 and BosR.

*In vivo*, BosR is essential for tick to mammal transmission and during mammalian infection but is not required for persistence in ticks, presumably because BosR is essential for the transcription of *rpoS* and regulates genes involved in the oxidative stress response [46,103,104,105,108,114,115]. Independent of its role as an activator of *rpoS* transcription, BosR has been predicted to regulate more than 100 genes outside of the RpoS regulon when cultivated at 37 °C to the mid-logarithmic phase of growth [74,103]. However, the functions of these genes are largely unknown, lending to the possibility that BosR regulates novel genes critical for *B. burgdorferi* virulence. Moreover, how BosR itself is regulated at the transcriptional and post-transcriptional levels is not well understood. Current work suggests that *bosR* transcription is driven from two putative promoters and that BosR activates its own transcription in a positive feedback loop [116]. Post-translationally, BosR protein levels may be regulated by RpoS [117]. Structurally, BosR consists of an N-terminal helix–turn–helix DNA binding domain and a C-terminal dimerization domain. Members of the Fur family typically bind a metal ion(s) at the S1 and S2 metal binding sites to control protein dimerization and determine regulatory function, respectively. Initially, it was unclear what metal served to coordinate BosR dimerization at the S1 site, but the purification of recombinant BosR identified that zinc is likely the only metal that associates with BosR via two CXXC motifs [105,118,119]. Interestingly, BosR lacks a complete S2 regulatory site. Several studies have been conducted to determine if BosR binding to DNA is affected by the presence of various metals including manganese, iron, zinc and copper; however, the results have been inconclusive [46,106,120]. To date, the signal responsible for shifting BosR between an “open” and “closed” conformation and consequently coordinating BosR-DNA binding remains elusive.

RpoS transcription and protein function are also regulated by PlzA, presumably through BosR [83]. First, *bosR* expression is positively regulated by PlzA at both the transcriptional and post-transcriptional levels; elevated BosR expression therefore leads to increased *rpoS* transcription [67]. More recently, it has also been suggested that BosR and PlzA coordinate RNAP-RpoS protein complex function to regulate the RpoS regulon [83]. Specifically, during mammalian infection, PlzA is unliganded due to low levels of c-di-GMP. In the absence of ligand-bound PlzA, BosR interacts with the RNAP-RpoS complex to transcribe the RpoS-upregulated genes required for mammalian infection and repress tick-phase genes (otherwise transcribed by RpoD). Conversely, in fed ticks, during transmission, c-di-GMP-bound PlzA binds to both the RNAP-RpoS complex and the RNAP-RpoD complex, resulting in the upregulation of RpoS-repressed tick-phase genes like *ospA* and *glp* [83].

In addition to the complex mechanisms of transcriptional activation, *rpoS* mRNA is regulated post-transcriptionally by a small non-coding RNA (DsrA_Bb_) and an RNA chaperone protein (Hfq). At low cell density, transcription from a canonical −35/−10 RpoD promoter produces an *rpoS* mRNA transcript that is 121 bp longer than the mRNA transcript produced from the −24/−12 RpoN promoter [121]. The extended 5’ end of this “long” transcript binds to DsrA_Bb_, allowing this *rpoS* mRNA to be post-transcriptionally regulated by DsrA_Bb_ in a temperature-dependent manner [121]. Specifically, DsrA_Bb_ binds directly to *rpoS* mRNA to expose the Shine–Dalgarno sequence and promote translation at a low cell density (~1-3 × 10^7^ cells per mL) when cultures are temperature-shifted from 23 °C to 37 °C [121]. The regulation of *rpoS* post-transcriptionally by DsrA_Bb_ is further controlled by the RNA chaperone protein, Hfq_Bb_ (BB0268), by the direct binding of Hfq_Bb_ to DsrA_Bb_ and *rpoS* mRNA [122]. Interestingly, Hfq (BB0268) promotes RpoS expression at both high and low cell densities, suggesting it also regulates *rpoS* independent of DsrA_Bb_. However, a recent study reported that BB0268 is not an Hfq homolog but a structural flagellar component (FlgV) in *B. burgdorferi* that modulates flagellar assembly [123]. Precisely how Hfq_Bb_ (BB0268) and DsrA_Bb_ effect *rpoS* translation remains to be elucidated.

Several studies have examined the contribution of another putative RNA binding protein, CsrA, on *rpoS* expression. The first studies concluded that CsrA regulates *rpoS* transcription and/or translation [124,125,126,127]. However, a subsequent group found that CsrA does not regulate *rpoS* or genes in the RpoS regulon including *ospC* or *dbpA* [128]. It is possible that differences in study designs have led to these discrepancies.

#### 4.1.2. Repression of rpoS Expression

While the activation of *rpoS* has been well studied in *B. burgdorferi*, the mechanisms of *rpoS* repression are less understood. BadR, a member of the ROK (repressor, open reading frame, kinase) family of transcriptional regulators, has been confirmed to bind to the *rpoS* promoter to repress transcription [129]. Subsequent work has established that BadR also binds the *bosR* promoter, an activator of *rpoS* transcription [130]. Therefore, it appears that BadR functions in multiple ways to regulate *rpoS* expression. First, by directly binding to the *rpoS* promoter to repress transcription; second, by repressing the transcription of the *rpoS* transcriptional activator protein, BosR.

Initial studies identified BadR as a repressor of *rpoS* and *bosR* transcription, presumably in the unfed tick when RpoS is “off” [129,130]. Interestingly, BadR is also required for mammalian infection when RpoS is turned “on,” suggesting that it serves a critical function independent of repressing *rpoS* [129,130]. Moreover, BadR is expressed under several conditions in vitro, providing additional evidence that the protein is important during multiple phases of the enzootic lifecycle [131]. In line with this hypothesis, global transcriptional analyses found that many genes are differentially expressed in a *badR* mutant when cells are cultivated under in vitro conditions, partially mimicking the mammalian host and unfed tick; moreover, the regulation of gene expression by BadR is growth phase dependent [129,131,132]. Genes encoding carbohydrate uptake and metabolism components have been consistently identified, suggesting that BadR functions as a key regulator of metabolism, possibly during multiple phases of the enzootic lifecycle. In fact, recent work demonstrated that BadR directly binds and regulates genes involved in glycerol uptake, substantiating BadR as a regulator of carbohydrate metabolism in *B. burgdorferi* [131,133]. However, most genes identified in the BadR regulon currently have no known function, making it difficult to discern the complete role of BadR in *B. burgdorferi* transcriptional regulation.

Structurally, BadR contains an N-terminal DNA-binding domain and C-terminal sugar binding domain [2,129,130]. In other bacterial pathogens, ROK family repressors typically bind a single sugar substrate, which induces a conformational change, releasing the protein from the promoter and allowing for the transcription of genes required for the utilization of that sugar. However, the binding of phosphorylated sugars to the BadR C-terminal sugar binding domain has been inconsistent, leaving open the possibility that another metabolite or small molecule coordinates its DNA-binding activity [129,130,133]. In addition to its function as a DNA binding protein, BadR may also serve as a post-transcriptional regulator [130]. Dual DNA and RNA binding activity has been observed for several other *B. burgdorferi* transcriptional regulators including SpoVG, BpuR, and PlzA [61,134,135,136,137]. BadR may also bind to mRNA, in addition to DNA, and regulates gene expression at the post-transcriptional level.

A second protein, BBD18, has been suggested to negatively regulate RpoS at the transcriptional and post-translational levels [24,138]. BBD18 is predicted to bind directly to *rpoS* promoter DNA to repress transcription. Post-translationally, BBD18 may bind directly to and destabilize RpoS protein, thus facilitating the transition of RpoS to an “off” state as *B. burgdorferi* enters and resides in the tick vector [24,25]. Furthermore, the post-translational repression of RpoS is indirectly regulated by the RNA polymerase binding protein, DksA, possibly by activating the expression of ClpAP/ClpXP proteases and/or BBD18, resulting in decreased RpoS protein [139,140]. The expression of RpoS protein is also significantly increased and decreased in Lon-1 and Lon-2 protease mutants, respectively, providing additional evidence that RpoS protein levels are directly influenced by protease activity, although the exact mechanisms remain unclear [141,142].

### 4.2. SpoVG

SpoVG protein was initially described in *Bacillus subtilis* as having a role in sporulation, but is ubiquitous in many bacteria including *Listeria monocytogenes*, *Staphylococcus aureus*, *Bacillus cereus,* and *B. burgdorferi* [143,144,145,146,147,148]. Structurally, SpoVG contains a C-terminal alpha-helix, which serves as a non-canonical nucleic acid-binding domain for binding DNA and RNA [134,135,148]. Interestingly, however, a consensus binding motif recognized by SpoVG has not been identified, lending to the possibility that the protein interacts with DNA targets based on DNA conformation versus a specific nucleic acid sequence.

The direct targets of SpoVG include its own DNA and mRNA, suggesting it functions as an auto-regulator of its own expression [135]. Additionally, SpoVG can bind directly to DNA regions upstream of glycerol uptake genes, indicating that the protein regulates genes that are critical for spirochete survival in unfed ticks [135]. Moreover, the deletion of SpoVG results in slower bacterial growth at 23 °C, indicating that the protein regulates genes involved in spirochete physiology and further substantiating a role for SpoVG in unfed ticks [135]. However, the complete SpoVG regulon, the requirement for SpoVG during the tick–mammal enzootic cycle, and the mechanism(s) by which SpoVG regulates it DNA and RNA targets are currently unknown.

### 4.3. BpuR, BpaB, and EbfC

BpuR, a PUR domain containing homodimer, is a transcriptional regulator capable of binding single- and double-stranded DNAs and RNA by a novel protein structure rather than a traditional DNA binding motif [149,150]. In the unfed tick, BpuR is turned “on” to repress the transcription of genes important for mammalian infection; BpuR is subsequently turned “off” during tick feeding to allow for the transcription of these genes. [136]. Post-transcriptionally, BpuR binds 5’mRNA ends to inhibit translation. Post-transcriptional targets include its own mRNA and numerous additional mRNA targets such as SodA, a superoxide dismutase that is essential during mammalian infection [136,151,152,153].

ParB proteins are found in other bacterial species and bind DNA to regulate plasmid replication and segregation. In *B. burgdorferi*, each *cp32* plasmid encodes a protein, BpaB, with homology to ParB. In addition to a role in plasmid maintenance, BpaB proteins act as gene repressors by occluding promoter sequences, primarily *erp* genes located on *cp32* plasmids [154,155,156]. Some evidence suggests that BpaB may also activate the transcription of genes including *nucP* and *ssbP*, encoding a nuclease and single-stranded DNA-binding protein, respectively [157].

EbfC proteins are well conserved amongst bacterial species, acting primarily in DNA organization as nucleoid-associated proteins [158,159,160,161]. In *B. burgdorferi*, EbfC forms a homodimer capable of directly binding DNA, but also forms tetramers and octamers that may function to connect separate DNA strands, consistent with a role in organizing DNA [160,162]. Additionally, EbfC has been proposed to play an indirect role in DNA replication through the DNA polymerase III subunit, DnaX [163,164].

In *B. burgdorferi*, *erp* genes are located on *cp32* plasmids and encode outer surface proteins that are capable of binding mammalian host plasminogen, laminin, glycosaminoglycans, and complement factors, thereby contributing to dissemination and overall infectivity [165,166,167,168,169,170]. All three transcriptional regulators, BpuR, BpaB, and EbfC, were initially identified for their role in regulating *erp* family operons [149,157,171,172]. Specifically, BpaB binds a sequence upstream of *erp* promoters and oligomerizes across the DNA, physically preventing gene transcription [165,171]. BpuR serves as a co-repressor with BpaB to repress *erp* transcription; however the precise mechanism is currently unknown [149].

In contrast, EbfC competes with BpaB for binding in the *erp* operator sequence, thereby acting as an anti-repressor of *erp* transcription [165,172]. *erp* expression is turned “off” in unfed ticks due to the increased expression of BpuR and BpaB repressors, and turned “on” during tick feeding and mammalian infection, when BpuR and BpaB expression decreases and EbfC levels increase [136]. Interestingly, EbfC levels increase as bacterial replication increases, suggesting that *erp* gene expression is directly linked to high bacterial replication rates, as seen in vivo during tick feeding [164,173].

### 4.4. The Stringent Response

It is well established that *B. burgdorferi* regulates its gene expression in response to various environmental conditions including temperature, pH, osmotic stress, and nutrient availability [3,16,17]. Throughout the enzootic lifecycle, spirochetes experience extended periods of nutrient starvation, leading to an array of transcriptomic changes. This highly conserved stress response is known as the stringent response. In *B. burgdorferi*, Rel_Bbu_ alone regulates the synthesis of intracellular levels of signaling molecules guanosine pentaphosphate and guanosine tetraphosphate [(p)ppGpp], which directly bind RNA polymerase to regulate gene expression [174,175,176]. Specifically, when spirochetes reside in the nutrient-starved unfed tick, the levels of (p)ppGpp rise. During tick feeding and in the nutrient-rich mammalian host, levels of (p)ppGpp decrease, leading to the differential expression of genes involved in replication, motility, morphology, and virulence [177].

(p)ppGpp-bound RNA polymerase exerts its transcriptional effects indirectly, through the regulatory protein DksA [178,179,180]. Investigations to the Rel_Bbu_ and DksA regulons identified significant overlap between these two, including the *glpF* and *glpK* genes that are essential for glycerol metabolism and spirochete persistence in ticks [177,181]. Additional genes in the Rel_Bbu_ regulon include *vlsE* (Vmp-like surface lipoprotein), *bosR*, *dbpBA,* and *bbk32*, which are important for mammalian host infection, as well as *cp32* bacteriophages and several small RNAs [177]. The same study also found that independent of its interplay with the stringent response, DksA regulates a unique subset of genes spanning several functional categories and located throughout the entire genome [180,182]. These genes encode proteins involved in basic cellular processes such as DNA replication, translation, and ribosomal subunits. A role for DksA independent of the stringent response has also been reported in other bacteria including *E. coli*, *Acinetobacter baumannii, Legionella pneumophila,* and *Yersinia enterocolitica*, to name a few [183,184,185,186,187]. Post-translationally, DksA and Rel_Bbu_ also regulate RpoS and consequently host adaptation, albeit the precise mechanism remains unknown [121,139,140,188].

Recently, DksA has been explored as a therapeutic target to treat drug-resistant bacteria such as *A. bumannii* due to its role in regulating biofilm development and other key virulence factors [189,190,191]. Likewise, DksA was proposed as a possible regulatory protein, modulating the persistence of antimicrobial-tolerant spirochetes. However, the exact role of DksA in antimicrobial resistance and its precise involvement in regulating virulence-associated genes have not been thoroughly defined in *B. burgdorferi*.

### 4.5. Small Regulatory RNAs

A variety of regulatory RNA species exist in nature and have been shown to be involved in many cellular functions, ranging from cell division to stress response and adaptation [192,193,194,195]. Generally, non-coding RNAs (ncRNAs) are characterized as small non-coding RNAs (sRNAs) or long non-coding RNAs (lncRNAs) if they are less than or greater than 200 nucleotides in length, respectively. *Trans*-acting sRNAs commonly function by direct base-pairing with target mRNA to alter its stability and/or translation efficiency, whereas *cis*-acting sRNAs frequently act as riboswitches and/or thermosensors that respond to environmental signals to regulate gene expression [196]. In addition to coordinating basic cellular processes, bacterial sRNAs have become recognized for their critical roles in regulating pathogenicity [197,198,199,200]. For example, sRNA *sprY* in *S. aureus* directly contributes to virulence by regulating RNAIII activity and reducing hemolysis [201,202]. In *E. coli*, *gcvB* has multifaceted roles regulating amino acid metabolism and oxidative stress response [203,204]. Likewise, a role for RNA regulators in *B. burgdorferi* gene regulation and pathogenicity is becoming increasingly clear [16].

In contrast to initial investigations, recent studies have identified between 317 and 1005 sRNAs in the *B. burgdorferi* genome, many of which may contribute to post-transcriptional regulation [205,206,207,208]. The prevalence of sRNAs in *B. burgdorferi* is not surprising considering the limited repertoire of transcriptional regulators, as well as, presumably, the need for additional gene regulatory mechanisms at the post-transcriptional level. Interestingly, the sRNA transcriptome varies significantly at 23 °C and 37 °C, suggesting specific sRNAs may be important during tick or mammalian phases of the enzootic lifecycle [205]. Although few RNA regulators have been characterized in *B. burgdorferi*, sRNAs have been predicted to regulate several critical virulence genes including *bosR*, *glpF*, and *hk1* [188]; in one case, they have been shown to be essential for *B. burgdorferi* mammalian infection [209]. Due to their diverse functions, abundance, and direct contributions to bacterial virulence, regulatory RNAs present a unique target for therapeutic interventions [196,210]. Therefore, it is essential to further elucidate the impact of RNA regulators on *B. burgdorferi* pathogenesis during the complete enzootic lifecycle.

## 5. Concluding Remarks

The mission for all microorganisms is to survive in nature. To maintain its tick–mammal infectious cycle, *B. burgdorferi* has evolved complex regulatory networks to coordinate environmental-responsive gene expression. Current data suggest that many regulatory proteins found in *B. burgdorferi* may have unconventional structures and functions, resulting in many unique regulatory mechanisms observed in this important human pathogen, which leaves numerous important questions open for future research. It is worth noting that gene regulation data are typically affected by experimental conditions, and many contradictory reports have been noted in the field. Further studies on gene regulation in *B. burgdorferi* are encouraged to not only resolve those controversies, but also to substantiate phenotypes observed in in vitro-grown spirochetes through in vivo analyses.

## Data Availability

No new data were created or analyzed in this study.
